# Special Issue “Molecular and Cellular Mechanisms of Epilepsy—3rd Edition”: Emerging Frontiers in Neuroinflammation, Network Remodeling, and Therapy

**DOI:** 10.3390/ijms262010020

**Published:** 2025-10-15

**Authors:** Aleksey V. Zaitsev

**Affiliations:** Sechenov Institute of Evolutionary Physiology and Biochemistry of RAS, 44, Toreza Prospekt, Saint Petersburg 194223, Russia; aleksey_zaitsev@mail.ru

## 1. Introduction

Epilepsy is one of the most common chronic neurological disorders, characterized by spontaneous recurrent seizures that cause substantial disability worldwide. While available antiseizure medications provide adequate seizure control for many patients, approximately 30% of those with temporal lobe epilepsy (TLE) develop pharmacoresistance [1,2]. This treatment gap underscores the urgent need for therapies that target underlying disease mechanisms rather than merely suppressing symptoms.

The conceptual framework of epilepsy has evolved substantially from earlier neuronal-centric views focused on channelopathies and synaptic imbalance. Contemporary research now frames epilepsy as a network disorder involving complex interactions between neuronal and non-neuronal elements [3]. A key paradigm shift has been the recognition of neuroinflammation as a fundamental mechanism in epileptogenesis. Specific inflammatory mediators—such as IL-1β, TNF-α, HMGB1, TGF-β, and prostaglandins—have been shown to directly modulate neuronal excitability, glial function, and blood–brain barrier integrity [4,5].

In parallel, the traditional concept of discrete epileptic foci has been superseded by a model of dynamic circuitopathy that emphasizes large-scale network reorganization [6,7]. Advanced methodologies, from optogenetics to connectomics, have demonstrated that ictogenesis emerges from distributed neural networks, which helps explain complex seizure propagation patterns and associated comorbidities [8].

This review synthesizes current knowledge across three interconnected domains that are reshaping epilepsy research: (1) the dynamic interplay between neuroinflammation and metabolic adaptations in epileptogenesis; (2) the mechanisms of circuit remodeling extending beyond the hippocampus; (3) the translation of these mechanistic insights into novel disease-modifying therapies. By integrating foundational principles with cutting-edge findings from this Special Issue, we aim to delineate the current molecular and cellular landscape of epilepsy and highlight critical directions for future investigation.

## 2. Neuroinflammation and Metabolic Adaptations in Epileptogenesis

The recognition of neuroinflammation as a core mechanism in epilepsy [4,9] has been extended by recent research revealing its tight interconnection with cerebral metabolism, forming a bidirectional signaling axis that critically influences epileptogenesis. This crosstalk operates through multiple pathways. For instance, pro-inflammatory cytokines like IL-1β and TNF-α can directly impair mitochondrial function by disrupting electron transport chain complexes and promoting reactive oxygen species (ROS) production [10,11]. Furthermore, neuroinflammatory signaling drives astrocytes toward a neurotoxic A1 phenotype, which loses normal homeostatic functions and contributes to metabolic dysregulation [12].

Astrocyte dysfunction is thus a critical nexus linking inflammation and metabolic impairment in epilepsy. Dysfunctional astrocytes exhibit impaired glutamate metabolism and disrupted gap junction coupling, which limits activity-dependent trafficking of energy metabolites and compromises the clearance of extracellular K^+^ and glutamate [13,14]. These alterations in astrocytic support directly promote neuronal hyperexcitability and create an energy-deficient state that favors seizure generation. The inflammatory milieu exacerbates this energy crisis via multiple mechanisms, including cytokine-mediated disruption of mitochondrial function and impairment of critical neuroglial metabolic interactions essential for network stability [4,15].

Conversely, metabolic stress itself potently activates neuroinflammatory pathways. Mitochondrial dysfunction and ROS overproduction can activate the NLRP3 inflammasome [16], creating a self-reinforcing cycle in which inflammation and metabolic dysfunction fuel each other, significantly accelerating epileptogenesis. The anti-seizure effects of the ketogenic diet may partly arise from breaking this cycle, by reducing glycolytic flux and decreasing NLRP3 inflammasome activation [17,18].

This metabolic-inflammatory interplay is central to the concept of metabolic exhaustion in chronic epilepsy. Recent evidence outlines a temporal trajectory of metabolic adaptation, from an acute compensatory response to chronic impairment. In this issue, Liotta et al. (2024) use proteomic kinetic modeling to demonstrate that while initial seizure activity upregulates oxidative phosphorylation to meet energy demands, chronic epilepsy is characterized by a significant decline in ATP production capacity [19]. This progressive metabolic deficit, associated with impaired mitochondrial function, contributes to neuronal hyperexcitability and lowers the seizure threshold, thereby representing a potential therapeutic target for disease modification.

Therapeutic strategies targeting neuroinflammatory pathways are advancing, with several showing promise in preclinical models [20]. Of particular interest are interventions that simultaneously address both inflammatory and metabolic components of epilepsy. The peroxisome proliferator-activated receptor (PPAR) system has emerged as a critical regulator of this crosstalk, modulating both lipid metabolism and anti-inflammatory signaling [21]. In this issue, Kovalenko et al. (2025) demonstrate that the PPARα agonist fenofibrate exerts region-specific effects during epileptogenesis, reducing anxiety-like behaviors and cortical expression of inflammasome components while differentially modulating glutamate receptor subunits across hippocampal subfields [22]. These findings underscore the promise and complexity of targeting metabolic-inflammatory pathways, as region-specific responses can influence both therapeutic efficacy and potential side effects.

In summary, the evidence confirms that neuroinflammation and metabolic dysfunction are fundamentally interconnected in epilepsy, forming a self-reinforcing cycle that drives disease progression. These processes influence each other bidirectionally, from cytokine-mediated mitochondrial impairment to metabolic stress-induced inflammatory activation. Consequently, future therapeutic development must employ integrated approaches that simultaneously target inflammatory signaling and metabolic pathways, while accounting for their temporal dynamics and regional specificity within brain circuits. Such strategies are essential to move beyond symptomatic control toward interventions that genuinely modify the disease course.

## 3. Circuit Reorganization and Distributed Dysfunction in Epilepsy

The conceptual understanding of epilepsy has thus expanded from a primary focus on hyperexcitable neuronal populations to encompass dysfunction across distributed neural networks. This paradigm shift acknowledges that seizure generation, propagation, and associated cognitive deficits are emergent properties of pathologically interacting large-scale brain circuits [8,23]. The impairment involves more than altered synaptic strength in canonical pathways like the hippocampal trisynaptic loop; it includes a fundamental reorganization of connectivity. This rewiring is characterized by the emergence of alternative pathways and the aberrant recruitment of extrahippocampal nodes, which collectively foster network hyperexcitability [24].

A critical manifestation of this process is the pathological reinforcement of typically subordinate neural pathways. A prime example is the direct temporoammonic pathway from entorhinal cortex layer III to the hippocampal CA1 region. Normally involved in contextual modulation, this pathway undergoes activity-dependent strengthening in epilepsy, forming a hyperexcitable bypass that circumvents the inhibitory gatekeeping function of the dentate gyrus [25].

Compelling evidence for this mechanism comes from Postnikova et al. (2024) in this Special Issue, who combined detailed morphological and electrophysiological analyses in a chronic epilepsy model [26]. Their work demonstrates a 2.5-fold increase in dendritic spine density in the *stratum lacunosum-moleculare* of CA1—the precise termination zone of the direct temporoammonic pathway. This substantial morphological change was accompanied by a twofold enhancement in the summation of synaptic responses upon pathway stimulation, indicating profound functional reinforcement. Importantly, this strengthening of the direct entorhinal input occurred while inputs from CA3 remained unaltered or diminished.

These findings provide concrete structural and functional support for the “breakdown of the dentate gate” hypothesis, which posits a failure of the dentate gyrus filter—a classic concept in epilepsy research [27,28]. Thus, epileptogenesis involves a specific pathological rerouting of information flow, creating a hyperexcitable shortcut within limbic circuits that likely contributes to the heightened epileptic activity in this model.

Circuit reorganization extends far beyond the hippocampus, implicating key extrahippocampal nodes in the governance of network excitability. The thalamus is increasingly recognized as a crucial regulator of cortical rhythms and a key participant in the oscillatory synchrony underlying various seizure types [29]. As highlighted in a recent comprehensive review, specific nuclei, including the anterior (ANT) and centromedian (CMT) thalamic nuclei, are critically involved in human epilepsy, where aberrant thalamocortical circuits are believed to underpin both epileptogenesis and ictal dynamics [30]. Via their diffuse reciprocal connections with the cortex, these nuclei exert a powerful influence over cortical activity, establishing them as central players in distributed epileptic networks.

Contemporary research aims to decipher the distinct molecular adaptations within these nodes that predispose the entire network to hyperexcitability. A prime example is the work of Kelemen et al. (2025) in this issue, who conducted a detailed mapping of neuronal calcium-binding protein 1 (NECAB1) in a chronic TLE model [31]. They revealed a striking bilateral upregulation of NECAB1-expressing neurons specifically within the paraventricular thalamic nucleus (PVT) and the amygdala—key hubs of the limbic network. This upregulation, modulated by the antiseizure medications levetiracetam and brivaracetam, likely represents a sustained, compensatory adaptation to hyperexcitability. Given that calcium-binding proteins are potent modulators of intrinsic excitability and short-term plasticity, such region-specific alterations indicate that individual network nodes acquire unique “epileptogenic signatures.” This advances the concept of a “molecular geography” for epileptic networks, where understanding the specific molecular landscape of each node is essential for predicting network behavior and identifying new therapeutic targets.

The functional consequences of this large-scale rewiring are complex and often counterintuitive, accounting for the variable efficacy of neuromodulation approaches. Optogenetic studies dissecting cell-type-specific contributions to seizure circuits clearly demonstrate this principle. Krook-Magnuson et al. (2013) showed that spontaneous hippocampal seizures can be suppressed by manipulating fundamentally different network elements: inhibiting excitatory principal cells was effective but so was activating a specific subpopulation of local GABAergic interneurons [32]. This proves that successful intervention depends on engaging the correct circuit element, not simply applying blanket inhibition or excitation.

The complexity of target engagement is further highlighted in large-scale networks. Wang et al. (2025) show that the centromedian thalamic nucleus (CMT), a key extrahippocampal node, exerts a powerful modulatory influence on hippocampal seizures [33]. They found that while a non-selective chemical lesion of the CMT abolished the initiation pattern of hippocampal seizures, selective chemogenetic suppression of only CMT pyramidal neurons shortened seizure duration without affecting the initial onset pattern. This indicates that distinct cell populations within a single node govern different facets of seizure dynamics—initiation versus maintenance—demonstrating that therapeutic outcomes depend critically on the specific neuronal subpopulation targeted. Consequently, the effect of an intervention is determined not only by its mechanism but by its precise cell-type and location within the reconfigured epileptic network. Targeting specific cell populations within critical network nodes therefore represents a promising strategy, grounded in a deeper understanding of the cellular and network organization of epilepsy.

## 4. Therapeutic Translation and Future Perspectives: The Dawn of Disease-Modifying and Precision Medicine

Translating the mechanistic insights detailed above—chronic neuroinflammation, metabolic exhaustion, and large-scale circuit remodeling—requires developing transformative therapies that move beyond symptomatic control toward genuine disease modification. For decades, epilepsy pharmacotherapy has been dominated by antiseizure medications (ASMs) that primarily suppress neuronal hyperexcitability. While these provide symptomatic relief for many patients, they largely fail to alter underlying epileptogenic processes and, critically, do not prevent epilepsy in at-risk individuals [34,35]. The field is now decisively shifting toward strategies that aim to arrest or reverse the disease course, including preventing epileptogenesis, guided by the principles of precision medicine [2,36].

Accurate diagnosis and stratification of patients based on the specific biological drivers of their disease are cornerstones of this new approach. Identifying robust biomarkers is paramount, enabling a shift from a syndromic to a mechanistic classification of seizures. Supporting this, Rider et al. (2024) demonstrate in this issue that the acute serum cortisol response effectively differentiates epileptic from psychogenic non-epileptic seizures [37]. Such biomarkers are crucial not only for initial diagnosis but also for identifying patient subgroups most likely to respond to specific, mechanism-targeted therapies—a core paradigm of precision medicine [36].

With accurate stratification, therapies can be directed against core pathological processes. The established role of neuroinflammation has validated it as a therapeutic target. Preclinical and early clinical studies have explored various anti-inflammatory strategies, including the interleukin-1 receptor antagonist anakinra, which shows promise in reducing seizure burden and modifying disease progression [38,39,40,41]. Simultaneously, the brain is increasingly viewed as part of a larger systemic network, with the gut–brain axis emerging as a potent modulator of excitability [42]. Interventions targeting the gut microbiome, such as specific probiotics or fecal microbiota transplantation, reduce seizure susceptibility in animal models, and the efficacy of the ketogenic diet may be partially mediated through microbial changes [43,44]. These approaches offer a non-invasive strategy for modulating the systemic environment that influences brain networks.

For patients with well-defined focal pathologies or specific genetic mutations, highly precise interventions are now in development. Gene therapy is at the forefront of this effort, advancing from proof-of-concept studies toward potential clinical application. As comprehensively reviewed by Mullagulova et al. (2024) in this issue, adeno-associated viral (AAV) vectors provide a powerful platform for targeted delivery of therapeutic transgenes to the epileptogenic focus [45]. Investigated strategies include overexpressing inhibitory neuropeptides like neuropeptide Y and galanin, enhancing potassium currents to dampen hyperexcitability, and silencing mutant alleles in genetic epilepsies [46,47,48,49]. This paradigm aims to achieve durable, circuit-specific rewiring of network activity, moving beyond pharmacological suppression toward potential one-time, curative interventions.

Therefore, the future lies in the convergence of these approaches. Epilepsy management will increasingly be guided by an integrated profile comprising the individual’s epileptogenic network signature, molecular diagnosis, systemic biomarker levels, and comorbidity profile. The principal challenge is to validate these strategies in robust clinical trials with disease modification as a primary endpoint and to address the disorder’s profound heterogeneity. Future progress depends on bridging the gap between acute animal models and chronic human epilepsy, validating broader panels of biomarkers for patient selection and monitoring, and integrating multi-omics data to build predictive disease models [7,50]. By viewing epilepsy through a multifaceted lens that interconnects inflammation, metabolism, networks, and systems biology, we pave the way for developing the sophisticated interventions required to prevent, cure, or effectively control this devastating disorder for all patients.
